# Assessing Osteoporosis Risk on Panoramic Radiography through Fractal Dimension Analysis of Mandibular Condyles in Postmenopausal Women

**DOI:** 10.1055/s-0046-1820075

**Published:** 2026-04-16

**Authors:** Sri Wigati Mardi Mulyani, Yunita Savitri, Alhidayati Asymal, Deny Saputra, Putri Alfa Meirani Laksanti, Norliza Ibrahim

**Affiliations:** 1 Department of Dentomaxillofacial Radiology, Faculty of Dental Medicine, Universitas Airlangga, Surabaya, Indonesia; 2 Master of Dental Health Sciences Study Program, Faculty of Dental Medicine, Universitas Airlangga, Surabaya, Indonesia; 3 Department of Oral and Maxillofacial Clinical Sciences, Faculty of Dentistry, Universiti Malaya, Kuala Lumpur, Malaysia

**Keywords:** fractal dimension, osteoporosis, panoramic radiography, postmenopausal women

## Abstract

**Objectives:**

The study aimed to analyze the trabecular bone structure pattern of the condylar bone on panoramic radiography using fractal dimension (FD) analysis and to identify the correlation between FD and bone mineral density (BMD) values to assess osteoporosis risk in postmenopausal women.

**Materials and Methods:**

This study involved 150 panoramic radiographs of postmenopausal women aged ≥50 years who were diagnosed with normal, osteopenia, and osteoporosis based on BMD values. FD analysis was performed on the condylar bone to establish standard FD values for normal, osteopenia, and osteoporosis conditions. The analysis was performed on the condylar bone using the box-counting method in ImageJ software to obtain FD values from skeletonized binary images of 30 × 30 pixel regions of interest (ROIs) on both condylar heads.

**Statistical Analysis:**

The normality of the data was assessed using the Shapiro–Wilk test, and correlations between variables were evaluated using Spearman's rank correlation analysis.

**Results:**

There was a significant positive correlation ( *p* < 0.001) between FD and BMD values, with moderate correlation strength ( *r* = 0.462 for ROI 1 and *r* = 0.518 for ROI 2). Higher BMD values were associated with higher FD values, and vice versa. Low FD values indicated low trabecular bone density (osteoporosis), while higher FD values reflected better trabecular bone density (normal).

**Conclusion:**

FD analysis of the mandibular condyles can serve as an indicator of osteoporosis. It complements BMD by quantifying trabecular bone structural changes, offering a method for early osteoporosis detection.

## Introduction

Osteoporosis is a systemic skeletal disorder marked by progressive loss of bone mass and deterioration of trabecular and cortical microarchitecture, resulting in increased bone fragility and fracture risk. 1 It is a major global health burden, affecting approximately 21.2% of women aged >50 years worldwide and contributing substantially to morbidity and mortality. 2 Indonesia is reaching 32.8% osteoporosis cases among postmenopausal women, ranking third in Southeast Asia and fifth globally. 3 Fragility fractures, particularly those of the spine, hip, and wrist, often lead to long-term disability, reduced quality of life, and increased healthcare costs. 1 Because structural degradation begins long before overt fractures occur, early detection of compromised bone quality in postmenopausal women is critical to prevent irreversible skeletal damage and improve clinical outcomes.

Dual-energy X-ray absorptiometry (DEXA) is the gold standard for evaluating bone mineral density (BMD) and diagnosing osteoporosis. However, limited accessibility, high cost, and low screening uptake, especially among asymptomatic individuals, restrict its widespread use in early detection. 4 These challenges highlight the need for simple, accessible, and opportunistic screening methods. Panoramic radiography offers several advantages in this context: it is widely available in dental practice, cost-effective, exposes patients to low radiation doses, and provides a broad view of maxillofacial structures. 5 Because panoramic imaging is routinely performed for dental examinations, it presents a practical platform for opportunistic osteoporosis screening.

A variety of radiomorphometric indices on panoramic radiographs have been proposed to identify low bone density, including the mandibular cortical index (MCI), mental index (MI), and panoramic mandibular index (PMI). These indices evaluate changes in cortical thickness or appearance and have shown potential in discriminating osteoporotic individuals. 6 However, their diagnostic performance is limited by operator-dependent interpretation, sensitivity to image quality, and a primary focus on cortical bone, whereas early osteoporotic changes mainly occur in the trabecular compartment. Consequently, radiomorphometric indices may not adequately reflect early microarchitectural deterioration. 7

Fractal analysis provides a quantitative method for characterizing complex trabecular patterns and has been used to evaluate bone microarchitecture in various anatomical sites. The fractal dimension (FD) reflects trabecular complexity and has shown promise in detecting microstructural changes associated with osteoporosis. 8 While previous studies have applied FD to different mandibular regions, 9 10 11 research specifically assessing the mandibular condyle, a location rich in trabecular bone and potentially sensitive to systemic skeletal changes, remains limited. Therefore, this study aims to analyze trabecular bone density of the mandibular condyle in postmenopausal women using FD analysis on panoramic radiographs, exploring its potential as an accessible adjunctive tool for early osteoporosis screening.

## Materials and Methods

### Study Design and Ethics Statement

This observational analytic study employed a retrospective design using secondary data from digital panoramic radiographs. Ethical approval was obtained from the Ethics Committee of the Faculty of Dental Medicine, Universitas Airlangga (No. 45/UN3.9.3/Etik/PT/2024). All procedures complied with institutional guidelines and the principles of the Declaration of Helsinki.

### Sample Selection

**Fig. 1:**
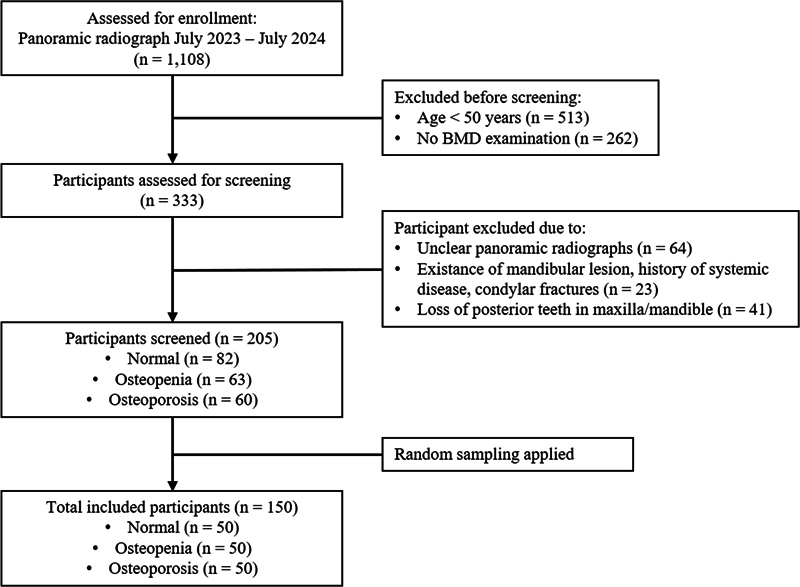
Sample selection procedure.

The minimum required sample size was calculated using the Lemeshow formula, 12 yielding a minimum of 30 samples. The sample selection procedure is shown in Fig. 1 , following the Strengthening the Reporting of Observational Studies in Epidemiology (STROBE) guideline. This study ultimately included 150 digital panoramic radiographs of postmenopausal women, consisting of 50 normal, 50 osteopenic, and 50 osteoporotic subjects, with image resolution of 1,976 × 976 pixels. All radiographs were obtained from the Radiology Unit of the Dental and Oral Hospital, Faculty of Dentistry, Universitas Airlangga, and were acquired using the Instrumentarium OP2pixels00 D-1 Digital Panoramic System (Instrumentarium Dental) with standardized exposure parameters of 70 kVp, 8 mA, and 12 seconds.

The radiographs were taken between July 2023 and July 2024. The inclusion criteria were as follows: (1) female patients aged ≥50 years who had undergone menopause; (2) confirmed diagnosis of normal, osteopenia, and osteoporosis based on DEXA-derived BMD T-scores; (3) clear visualization of both right and left mandibular condyles; (4) radiographs meeting standard panoramic image quality criteria; and (5) the absence of mandibular pathology or lesions that could affect bone structure or masticatory function. Patients with a history of systemic disease or condylar fractures affecting bone structure, horizontal/vertical distortion in panoramic images, or loss of posterior teeth in the maxilla or mandible were excluded from this study.

### Fractal Dimension Measurements

All panoramic radiographs were imported into ImageJ v1.52 for fractal analysis. The regions of interest (ROIs) were manually placed on the right and left mandibular condylar heads, each defined as a standardized 30 × 30-pixel square. ROI.1 represented the trabecular region of the right condyle, and ROI.2 represented the corresponding region on the left side. Each ROI was cropped and duplicated for subsequent processing.

**Fig. 2:**
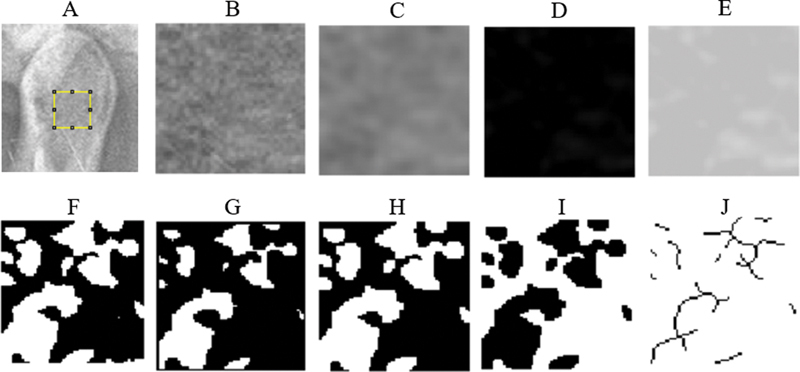
Fractal analysis process. ( **A** ) A 30 × 30 pixel ROI was cropped from the condyle. ( **B** ) The cropped image. ( **C** ) Application of the Gaussian blur filter. ( **D** ) Subtraction of the blurred image from the original image. ( **E** ) Addition of a grayscale value of 128 to each pixel location. ( **F** ) Binarization to visualize bone marrow spaces and trabeculae. ( **G** ) Erosion process. ( **H** ) Dilation process. ( **I** ) Inversion. ( **J** ) Skeletonization.

The duplicated images were processed by applying a Gaussian blur (sigma = 2) first to reduce grayscale fluctuations caused by overlapping structures and soft-tissue noise. The blurred output was then subtracted from the original ROI, producing an enhanced image that preserved trabecular outlines while suppressing background variations. The resulting image was then added a grayscale value of 128 to each pixel location and converted into binary format, where trabecular structures appeared black and marrow spaces appeared white. To minimize noise, the binary image underwent three iterations of erosion and dilation, yielding a cleaner representation of the trabecular pattern. In the final stage, the processed binary image was inverted and skeletonized, producing a simplified trabecular network suitable for fractal computation. These image-processing steps are illustrated in Fig. 2 .

FD values were calculated using the box-counting method in ImageJ (“Fractal Box Counter” tool). The algorithm divides the skeletonized ROI into progressively smaller box sizes (2, 3, 4, 6, 8, 12, 16, 32, and 64 pixels) and computes the logarithmic relationship between box size and the number of occupied boxes. The slope of the regression line obtained from this plot represents the FD value, reflecting the complexity of the trabecular structure. FD measurements were evaluated by three observers with more than 20 years of experience (S.W.M.M. and Y.S.) and more than 10 years of experience (D.S.) in oral and maxillofacial radiology. Inter-rater reliability was then assessed to determine the level of agreement between observers.

### Statistical Analysis

FD values were treated as continuous variables. Inter-rater reliability among the three observers was assessed using the intraclass correlation coefficient (ICC). A two-way random-effects model based on single measurements and absolute agreement was applied to evaluate the level of agreement among raters. ICC values were reported with 95% confidence intervals and interpreted as follows: <0.50, poor; 0.50 to 0.75, fair; 0.75 to 0.90, good; and >0.90, excellent agreement. 13

Normality of the data was then examined using the Shapiro–Wilk test prior to further analyses. Because BMD T-scores are continuous variables, their correlation with FD values was analyzed using Spearman correlation due to the normality of the data distribution. The osteoporosis status (normal, osteopenia, and osteoporosis) is an ordinal variable; therefore, its relationship with FD values was also assessed using Spearman's rank correlation. In addition to the correlation test, differences in FD values among the normal, osteopenia, and osteoporosis groups were analyzed using the Kruskal–Wallis test, followed by post-hoc pairwise comparisons with the Dwass–Steel–Critchlow–Fligner test. All statistical analyses were performed using Statistical Package for the Social Sciences (SPSS) Statistics version 26, with the significance level set at *p* < 0.05.

## Results

The distribution of age and BMD T-scores among the 150 samples, consisting of 50 samples in each group, is presented in Table 1 . All groups showed a relatively similar age distribution, indicating that screening should be conducted early, particularly around the age of 50 years. The ICC test among observers showed a *p* -value of 0.031, indicating statistically significant agreement between raters ( *p* < 0.05). The ICC value of 0.844 indicates good agreement.

**Table 1:** Distribution of data

		Normal ( *n* = 50)	Osteopenia ( *n* = 50)	Osteoporosis ( *n* = 50)
Age (years)	Range	51–82	51–80	51–89
	Mean ± SD	63.67 ± 8.07	62.16 ± 7.88	62.98 ± 8.19
T-Score BMD	Range	(−0.9)–3	(−2.4)–(−1.0)	(−4.5)–(−2.5)
	Mean ± SD	0.16 ± 0.89	(−1.66) ± 0.41	(−3.04) ± 0.44

In Table 2 , the results of the normality test show that the BMD T-score values and the FD values of the right and left condylar bone (ROI.1 and ROI.2) were not normally distributed, as indicated by a *p* -value <0.05. Since the data are not normally distributed, further data analysis was conducted using a correlation test, followed by the Spearman correlation test between the BMD T-score and the FD measurement values.

**Table 2:** Normality test result

Variable	*p* -Value	Interpretation
T-Score-BMD	0.006	Not normally distributed
ROI 1	0.015	Not normally distributed
ROI 2	< 0.001	Not normally distributed

Note: *p* -Value > 0.05.

In Table 3 , the results of the correlation test show a significant correlation between the BMD T-score and ROI.1, with a moderate correlation strength ( *r* = 0.462) and a positive relationship. Similarly, there was a significant correlation between the BMD T-score and ROI.2, with a moderate correlation strength ( *r* = 0.518) and a positive relationship. Meanwhile, for osteoporosis status, there was a significant correlation between osteoporosis status and both ROI.1 and ROI.2, with a moderate correlation strength for ROI.1 ( *r* = –0.483) and ROI.2 ( *r* = –0.526), showing a negative relationship.

**Table 3:** Correlation test result

	ROI 1		ROI 2	
	*p* -Value	*r*	*p* -Value	*r*
T-Score BMD	<0.001	0.462	<0.001	0.518
Osteoporosis status	<0.001	−0.483	<0.001	−0.526

The comparison test result conducted with Kruskal–Wallis showed a significant difference between normal, osteopenia, and osteoporosis ( *p* <0.001) in both right and left condyles. The post-hoc analysis was then evaluated and also showed a significant difference between normal and osteopenia, normal and osteoporosis, osteopenia and osteoporosis (all *p* <0.05), both in the right and left condyles. These results are shown in Table 4 .

**Table 4:** Post-hoc test result

Pairwise comparison	*p* -Value of right condyle	*p* -Value of left condyle
Normal and osteopenia	0.003	<0.001
Normal and osteoporosis	<0.001	<0.001
Osteopenia and osteoporosis	0.011	0.028

**Fig. 3:**
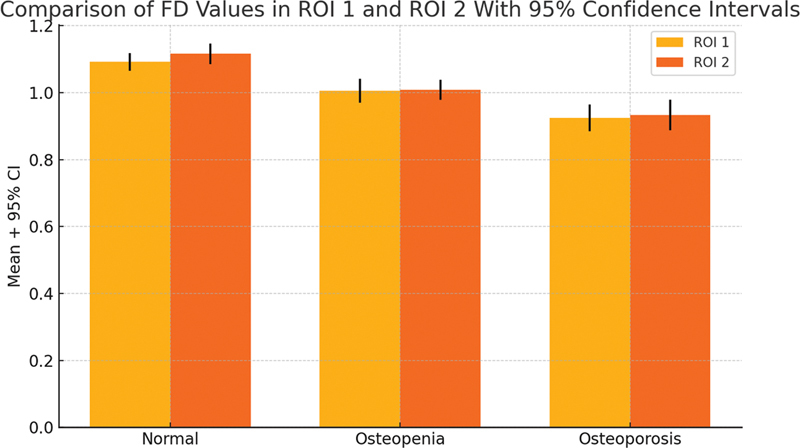
Fractal dimension (FD) values with a 95% confidence interval (CI) across bone conditions (normal, osteopenia, and osteoporosis) for ROI.1 and ROI.2.

The mean results of trabecular bone in the mandibular condyle showed that the average FD values of the right condylar trabecular bone (ROI.1) and the left condylar trabecular bone (ROI.2) under normal conditions were higher than those in osteopenia and osteoporosis ( Table 5 ). Similarly, the estimated mean FD values showed that the mean FD under normal conditions was higher than in osteopenia or osteoporosis. Fig. 3 illustrates the comparison of FD values with standard deviations across bone conditions for ROI 1 and ROI 2.

**Table 5:** Estimated parameters of ROI.1 and ROI.2 with a 95% confidence interval

Status	ROI	Mean ± SD	Lower bound	Upper bound	Estimated mean
Normal	ROI 1	1.0917 ± 0.0964	1.0650	1.1184	1.0650 < µ < 1.1184
	ROI 2	1.1157 ± 0.1100	1.0852	1.1462	1.0852 < µ < 1.1462
Osteopenia	ROI 1	1.0059 ± 0.1278	0.9705	1.0413	0.9705 < µ < 1.0413
	ROI 2	1.0085 ± 0.1089	0.9783	1.0387	0.9783 < µ < 1.0387
Osteoporosis	ROI 1	0.9246 ± 0.1454	0.8843	0.9649	0.8843 < µ < 0.9649
	ROI 2	0.9330 ± 0.1651	0.8872	0.9788	0.8872 < µ < 0.9788

## Discussion

This study investigated the relationship between BMD and the FD values of the mandibular condyle in postmenopausal women using panoramic radiographs. FD analysis is a statistical method that uses fractal mathematics to quantify complex shapes and structural patterns in bone texture. 14 This study found a significant positive correlation between the BMD T-score and FD values in both the right and left mandibular condyles. This means that as bone density (BMD) increases, the complexity of the trabecular structure also increases. The BMD T-score measures bone density, while the FD value quantifies the complexity of the trabecular bone pattern. Therefore, a higher FD value indicates a denser and more finely structured trabecular bone. This finding aligns with Amuk et al., who confirmed that FD significantly correlates with bone's physical properties. 15 Trabecular bone is more metabolically active than cortical bone, making it more sensitive to structural changes and thus a better site for such analysis. 16 A low FD value suggests more voids and poorer bone quality, whereas a high FD value indicates a denser, more complex structure. Thus, FD analysis performed on panoramic radiographs has a significant potential to evaluate bone density and microarchitectural quality. 14

Osteoporosis status was categorized as 1 (normal), 2 (osteopenia), and 3 (osteoporosis). In this study, a negative correlation was observed between osteoporosis and bone density in both the right (ROI 1) and left (ROI 2) condyles. The results demonstrated a direct relationship between FD values and bone quality. A high FD value is associated with a dense trabecular bone, whereas a low FD reflects the sparse trabecular structure of osteoporotic bone. Similarly, Calciolari et al reported that higher-density trabecular bone, characterized by uniform distribution, signifies better bone quality and lower fracture risk, while lower density reflects the uneven structure of osteoporosis and higher risk of fracture. 17 Several previous studies reported inconsistent associations between FD and osteoporosis. 18 19 While some observed increased FD in osteoporotic bone, 20 21 others reported a decrease in FD values. 22 23 Among various approaches, the box-counting method is the most frequently applied. Thus, it was utilized for the present study.

The moderate correlation observed in this study can be explained by the fundamental differences between FD analysis and DEXA. DEXA quantitatively measures BMD, reflecting the mineral content of bone, 4 whereas FD evaluates the structural complexity and spatial organization of trabecular bone. 8 Although mineral density and trabecular architecture are biologically related, they represent distinct components of bone quality. 17 Therefore, changes in trabecular pattern complexity may not always proportionally mirror the magnitude of mineral density reduction measured by DEXA. Consequently, FD analysis cannot be considered a primary diagnostic standard for osteoporosis but rather a potential adjunctive screening tool. Individuals identified as at risk through the FD assessment should undergo further evaluation using BMD measurement to confirm diagnosis and determine appropriate management.

Yurtoglu et al stated that osteopenia is a condition characterized by decreased BMD, in which structural changes are not yet clearly visible and bone density remains within the normal range. 24 However, the results of the Kruskal–Wallis test in the present study ( Table 4 ) demonstrated a statistically significant difference between the normal and osteopenia groups. These findings indicate that although osteopenia presents minimal observable changes in trabecular bone structure, panoramic radiography may still detect osteopenia conditions through FD analysis.

Additionally, the age distribution of the sample also indicated that the mean age in the osteopenia and osteoporosis groups was slightly younger than that of the normal group. This condition may be influenced by various factors, including lifestyle, nutritional status, and deleterious habits. Barak reported that differences in trabecular bone structure patterns may be attributed to several uncontrollable factors, such as unilateral mastication, tooth loss, nutritional factors, and parafunctional habits like bruxism. 25

The current study demonstrated that FD analysis on panoramic radiographs can serve as an alternative method for assessing mandibular condyle trabecular bone quality to indicate osteoporosis. These results suggest that the architectural deterioration of trabecular bone provides a valuable diagnostic indicator for osteoporosis and a reliable predictor of fracture risk. However, this study did not compare FD with other established panoramic indices such as MCI, MI, or PMI, as the primary objective was to focus specifically on FD measurement as an initial exploratory analysis of condylar trabecular patterns. This study has several limitations, including the use of low resolution of panoramic radiographs, a small sample size, uncontrolled confounding factors like detrimental habits, and the absence of additional clinical variables such as body mass index (BMI), disease duration, and lifestyle factors that may influence overall skeletal condition. Future research should utilize larger datasets with higher-resolution images and incorporate additional clinical and metabolic variables to better evaluate their influence on trabecular bone changes and improve the generalizability of the findings. FD analysis on panoramic radiographs can be used as an alternative method to evaluate trabecular bone quality in the mandibular condyle, thereby aiding in the identification of osteoporosis and fracture risk.

## Conclusion

FD analysis using panoramic radiography can be used as an alternative method to assess the quality of trabecular bone in the mandibular condyle, serving as an indicator for osteoporosis and fracture risk.
